# Respiratory Dysfunction in Epileptic Encephalopathies: Insights and Challenges

**DOI:** 10.7759/cureus.46216

**Published:** 2023-09-29

**Authors:** Muhammad Ali Khan, Shah Dev, Maneesha Kumari, FNU Mahak, Ahmed Umair, Maham Rasool, Aneesha Kumari, FNU Payal, Uttam Panta, FNU Deepa, Giustino Varrassi, Mahima Khatri, Satesh Kumar

**Affiliations:** 1 Medicine, Shaikh Khalifa Bin Zayed Al-Nahyan Medical and Dental College, Lahore, PAK; 2 Medicine, Jinnah Sindh Medical University, Karachi, PAK; 3 Medicine, Peoples University of Medical and Health Sciences for Women, Shaheed Benazirabad, PAK; 4 Medicine, Jinnah Postgraduate Medical Center, Karachi, PAK; 5 Medicine, Fatima Memorial College of Medicine and Dentistry, Lahore, PAK; 6 Medicine, King Edward Medical University (KEMU), Lahore, PAK; 7 Medicine, Shaheed Mohtarma Benazir Bhutto Medical University, Larkana, PAK; 8 Medicine, Chitwan Medical College, Bharatpur, NPL; 9 Medicine, Ghulam Muhammad Mahar Medical College, Sukkur, PAK; 10 Pain Medicine, Paolo Procacci Foundation, Rome, ITA; 11 Medicine and Surgery, Dow University of Health Sciences, Karachi, PAK; 12 Medicine and Surgery, Shaheed Mohtarma Benazir Bhutto Medical College, Karachi, PAK

**Keywords:** diagnostic challenges, neurobiological mechanisms, breathing abnormalities, seizures, respiratory dysfunction, epileptic encephalopathies

## Abstract

Epileptic encephalopathies constitute a group of severe epileptic disorders characterized by intractable seizures and cognitive regression. Beyond the hallmark neurological manifestations, these disorders frequently exhibit associated respiratory dysfunction, which is increasingly recognized as a critical aspect of their pathophysiology. Respiratory abnormalities in epileptic encephalopathies encompass a spectrum of manifestations, ranging from subtle alterations in breathing patterns to life-threatening events such as apneas and hypoventilation. These respiratory disturbances often occur during seizures, the interictal period, or even persist chronically, leading to significant morbidity and mortality. We explore the varied clinical presentations and their implications on patient outcomes, emphasizing the need for heightened awareness among clinicians. This review unravels the intricate mechanisms linking epilepsy and respiratory dysfunction. GABAergic and glutamatergic imbalances, alterations in central respiratory centers, and abnormal autonomic control are among the key factors contributing to respiratory disturbances in these patients. We elucidate the neurobiological intricacies that underlie these processes and their relevance to therapeutic interventions. Accurate diagnosis of respiratory dysfunction in epileptic encephalopathies is often hindered by its diverse clinical phenotypes and the absence of routine screening protocols. We scrutinize the diagnostic hurdles, highlighting the necessity of comprehensive respiratory assessments in managing these patients. Timely recognition of respiratory issues may guide treatment decisions and mitigate complications. Management of respiratory dysfunction in epileptic encephalopathies is complex and necessitates a multidisciplinary approach. We explore various therapeutic modalities, including antiepileptic drugs (AEDs), ventilatory support, and novel interventions like neuromodulation techniques. The review emphasizes the individualized nature of treatment strategies tailored to each patient's specific needs. In conclusion, this narrative review offers a comprehensive overview of respiratory dysfunction in epileptic encephalopathies, shedding light on its clinical importance, underlying mechanisms, diagnostic challenges, and therapeutic considerations. By addressing these insights and challenges, we hope to inspire further research and innovation to enhance the care and outcomes of patients with epileptic encephalopathies.

## Introduction and background

Epileptic encephalopathies represent a perplexing and devastating group of neurological disorders that profoundly impact the lives of affected individuals and their families. These conditions, often diagnosed in infancy or early childhood, are characterized by the convergence of severe epilepsy and cognitive regression, leading to a relentless cycle of seizures and neurodevelopmental decline [1]. Epileptic encephalopathies comprise a spectrum of rare and severe epileptic syndromes characterized by intractable seizures and neurodevelopmental deterioration. These disorders deviate from the typical course of epilepsy by featuring a dynamic interplay between epileptic activity and cognitive regression. Prominent examples within this category include Dravet syndrome, Lennox-Gastaut syndrome (LGS), and Ohtahara syndrome [2]. Dravet syndrome, formerly known as severe myoclonic epilepsy of infancy (SMEI), is one of the quintessential epileptic encephalopathies. Dravet syndrome typically emerges during the first year of life with seizures triggered by fever, often progressing to recurrent and intractable seizures of varying types, including myoclonic, tonic-clonic, and absence seizures [1]. As the syndrome advances, patients experience a gradual regression of cognitive and motor functions, leading to profound intellectual disability. Dravet syndrome is primarily associated with mutations in the *SCN1A* gene, encoding the voltage-gated sodium channel Nav1.1, highlighting the genetic underpinnings of epileptic encephalopathies [2][3]. LGS represents another archetypal epileptic encephalopathy characterized by multiple seizure types, including tonic, atonic, and atypical absence seizures. LGS typically manifests between the ages of one and eight and is often refractory to conventional antiepileptic drugs (AEDs) [4]. Cognitive decline and behavioral disturbances are hallmarks of the syndrome, leading to significant impairments in daily functioning. The etiology of LGS is multifaceted, with various structural, genetic, and metabolic factors contributing to its pathogenesis [5]. Ohtahara syndrome, on the other hand, presents a distinctive profile within the realm of epileptic encephalopathies. This syndrome emerges within the first few weeks of life and is characterized by intractable seizures, including tonic spasms, focal seizures, and multifocal epileptiform activity on electroencephalogram (EEG) [6]. Cognitive regression is rapid and profound, with most affected individuals developing severe intellectual disabilities. Ohtahara syndrome often arises from structural brain abnormalities or genetic mutations that disrupt normal brain development [7]. These brief insights into three prominent epileptic encephalopathies exemplify the heterogeneous nature of this group of disorders, underscoring the commonality of refractory seizures and cognitive decline as defining features [1,4,6]. While the neurological manifestations of epileptic encephalopathies have been the primary focus of research and clinical care, recognizing respiratory dysfunction as a critical component of these disorders has gained momentum. Respiratory abnormalities in epileptic encephalopathies encompass a spectrum of manifestations. Many individuals with epileptic encephalopathies exhibit altered breathing patterns, such as periodic breathing or irregular respirations [5]. Central apneas, characterized by temporary cessation of breathing, can occur during seizures or independently, contributing to oxygen desaturation and potential respiratory compromise [3]. Hypoventilation, often accompanied by hypercapnia (elevated carbon dioxide [CO_2_] levels), may develop chronically, exacerbating respiratory challenges [1]. The significance of studying respiratory dysfunction in the context of epileptic encephalopathies arises from several key considerations: respiratory abnormalities have emerged as substantial contributors to the morbidity and mortality associated with epileptic encephalopathies. Seizure-related respiratory events can lead to oxygen desaturation, cardiac arrhythmias, and, in severe cases, sudden unexpected death in epilepsy (SUDEP) [1,4]. Additionally, chronic hypoventilation may contribute to the accumulation of CO_2_ (hypercapnia) and the development of chronic respiratory failure, further compounding the clinical challenges faced by patients and healthcare providers [6]. The relationship between epilepsy and respiratory function is bidirectional and complex. Seizures can directly impact respiration, leading to alterations in breathing patterns, apneas, or oxygen desaturation [4]. Conversely, respiratory dysfunction, such as chronic hypoventilation, can influence seizure susceptibility and exacerbate the severity of seizures [2]. Understanding this dynamic interplay is critical for optimizing patient care. Diagnosing and characterizing respiratory dysfunction in individuals with epileptic encephalopathies pose significant challenges. Respiratory abnormalities can be subtle, may not always occur during routine clinical evaluations, and may overlap with other clinical features, such as autonomic dysfunction [3]. These diagnostic intricacies underscore the need for comprehensive assessments beyond traditional neurological examinations. This narrative review aims to provide a comprehensive examination of respiratory dysfunction in the context of epileptic encephalopathies. This review aspires to achieve the following objectives: By synthesizing existing research findings, clinical observations, and emerging insights, this review aims to shed light on the multifaceted nature of respiratory dysfunction in individuals with epileptic encephalopathies. It will delve into the various clinical presentations of respiratory abnormalities, the underlying mechanisms contributing to these disturbances, the diagnostic challenges clinicians face, and the therapeutic considerations available. The review will explore the intricate interplay between epilepsy and respiratory function, emphasizing the bidirectional nature of their relationship. It will elucidate the neurobiological mechanisms connecting epilepsy and respiratory disturbances, providing a deeper understanding of how these two domains intersect. Given the diagnostic complexities surrounding respiratory dysfunction in epileptic encephalopathies, this review will address the challenges clinicians face in identifying and characterizing these abnormalities. It will also explore potential solutions and strategies for comprehensive respiratory assessments that can aid in timely diagnosis and intervention. Management of respiratory dysfunction in the context of epileptic encephalopathies requires a multidisciplinary approach. This review will explore various therapeutic modalities, including AEDs, ventilatory support options, and emerging interventions, such as neuromodulation techniques. It will emphasize the individualized nature of treatment strategies tailored to each patient's specific needs. The broader implications of respiratory abnormalities on the clinical course and prognosis of individuals with epileptic encephalopathies will be explored. Case studies and clinical evidence will be employed to illustrate the impact of respiratory dysfunction on patient outcomes. Through this comprehensive review, we seek to provide a holistic understanding of the interplay between epileptic encephalopathies and respiratory dysfunction, offering valuable insights for healthcare professionals, researchers, and caregivers. By comprehensively assessing the significance of respiratory issues and their challenges, we aim to inspire further research and innovation to enhance the quality of life and clinical care for individuals grappling with these devastating conditions.

## Review

Methodology

The methodology employed for this narrative review involved a systematic and comprehensive search of electronic databases to identify relevant articles. The following databases were utilized for this search: PubMed/MEDLINE, Scopus, Web of Science, and Embase. The primary objective was to gather a comprehensive collection of literature on respiratory dysfunction in the context of epileptic encephalopathies. The search strategy was meticulously designed to capture a broad spectrum of articles. It encompassed a combination of keywords and controlled vocabulary terms relevant to epileptic encephalopathies and respiratory dysfunction. Terms such as "epileptic encephalopathies," "breathing abnormalities," "hypoventilation," "apnea," and "seizures" were utilized in various combinations. Boolean operators, such as "AND" and "OR," were employed to refine the search and optimize its sensitivity. Significantly, the search strategy was adapted to suit each database's specific requirements and indexing systems. No restrictions were imposed on the publication date, ensuring that historical and contemporary literature were considered. Articles had to meet predefined criteria to be eligible for inclusion in this narrative review. First, relevance was a paramount criterion. Articles are needed to directly address respiratory dysfunction in the context of epileptic encephalopathies or closely related syndromes. This criterion encompassed various article types, including original research studies and comprehensive review articles. Second, human studies were exclusively considered, focusing on the clinical implications and challenges faced by individuals with epileptic encephalopathies. Third, full-text availability was a requirement for inclusion to ensure complete and detailed information availability. Exclusion criteria were also established to maintain the focus and relevance of the review. Articles that deviated from the central theme of respiratory dysfunction in epileptic encephalopathies were excluded. Non-English language articles were omitted due to linguistic limitations. Animal studies were also excluded, as the primary aim was to explore the clinical aspects of respiratory dysfunction in humans. Additionally, duplicate publications or studies that substantially overlapped with included articles were excluded to avoid redundancy. Following the initial search and retrieval of articles, a two-stage screening process was employed. The first stage involved a review of titles and abstracts by two independent reviewers to assess their relevance to the review's objectives and their alignment with the inclusion criteria. Discrepancies or uncertainties were resolved through discussion and consensus between the reviewers. In the second stage, full-text articles were obtained for those abstracts that met the initial screening criteria. Each article was examined meticulously to determine its suitability for inclusion in the narrative review. Once the articles were selected for inclusion, a systematic approach to data synthesis was employed. Information of interest encompassed various facets of respiratory dysfunction in epileptic encephalopathies, including clinical manifestations, underlying mechanisms, diagnostic challenges, therapeutic interventions, and clinical outcomes. Data were extracted, organized, and summarized, focusing on key findings and insights from the selected articles. The narrative approach was employed to present the information coherently and contextualized, allowing for the integration of diverse perspectives and clinical observations. It is important to note that, due to the narrative nature of this review, a formal quality assessment or risk of bias analysis was not conducted. The articles were included based on their relevance to the review's objectives and their contribution to understanding respiratory dysfunction in the context of epileptic encephalopathies. This approach was deemed appropriate given the overarching goal of synthesizing existing literature to provide comprehensive insights and address the challenges posed by respiratory dysfunction in this clinical context.

Clinical manifestations of respiratory dysfunction

The presence of respiratory dysfunction within the framework of epileptic encephalopathies involves a broad range of anomalies that can substantially impact the quality of life for those affected. Comprehending these clinical manifestations is of utmost importance for clinicians and caregivers, as they exhibit a range of severity and frequently contribute to the overall intricacy of these disorders. This study examines various types of respiratory abnormalities that have been observed. Irregularities in respiratory patterns are commonly noticed in persons diagnosed with epileptic encephalopathies. These irregularities may present as irregular, superficial, or complex respiration occurrences, occasionally alternating with regular respiration intervals. These patterns may exhibit greater prominence during sleep but can also manifest during awake periods [8]. Apneas, defined as momentary interruptions in breathing, are frequently observed in persons diagnosed with epileptic encephalopathies. Apneas may manifest during or immediately after seizures, frequently as a component of the postictal phase. Central apneas are a significant concern due to the failure of the central respiratory centers to begin breathing [3]. Oxygen desaturation may be observed in conjunction with bradycardia, contributing to the overall clinical complexity. Chronic hypoventilation is characterized by insufficient ventilation, resulting in excessive CO_2_ levels, known as hypercapnia. This respiratory anomaly is observed in specific individuals with epileptic encephalopathies [3]. The manifestation of this condition can be observed through either shallow or slow breathing, and its effects may be particularly noticeable during periods of sleep. Persistent hypoventilation can lead to a chronic increase in CO_2_ levels, adversely affecting multiple physiological systems and general well-being. Certain persons diagnosed with epileptic encephalopathies may encounter acute respiratory distress during or after seizures. The manifestation of this condition includes heightened respiratory effort, observable inward chest or sternum movement known as retractions, and cyanosis, characterized by a bluish tint in the skin or mucous membranes. These symptoms serve as indicators of inadequate oxygen supply [4]. Although not a primary clinical manifestation, it is crucial to acknowledge the possible life-threatening implications of respiratory failure in individuals with epileptic encephalopathies. Sudden unexpected death in epilepsy (SUDEP) is an infrequent yet profoundly distressing event marked by the abrupt and inexplicable demise of an individual diagnosed with epilepsy, frequently occurring during periods of sleep. The prevailing belief is that this phenomenon is attributable to a confluence of variables, encompassing both seizure-induced respiratory failure and cardiac abnormalities [5].

The Correlation Between Seizures and Respiratory Dysfunction

The intricate and bidirectional link between seizures and respiratory failure in epileptic encephalopathies contributes to the complexity of these conditions. Seizures have the potential to affect respiratory function directly. During convulsive seizures, there is a temporary paralysis of the respiratory muscles, which can result in hypoventilation or apnea. This issue is particularly problematic in the context of tonic-clonic seizures, as individuals may experience temporary cessation of breathing [9]. Additionally, it should be noted that the postictal state after a seizure can also be linked to abnormal breathing patterns and apnea episodes, which can exacerbate respiratory dysfunction [7]. On the other hand, chronic respiratory dysfunction, such as hypoventilation and hypercapnia, can heighten the vulnerability to seizures. Increased CO_2_ levels can modify the excitability of neurons, resulting in a reduced seizure threshold. This might result in a higher occurrence and greater intensity of seizures in those affected [8]. This reciprocal association emphasizes the significance of managing respiratory problems in epileptic encephalopathies.

The Influence on Patient Quality of Life and Prognosis

Respiratory dysfunction in persons with epileptic encephalopathies significantly affects their quality of life and long-term prognosis. The presence of abnormal breathing patterns, apneas, and episodes of respiratory distress in persons with epileptic encephalopathies can profoundly impact their everyday functioning. Respiratory disturbances can potentially disrupt sleep patterns, resulting in chronic sleep deprivation for both the individuals experiencing these disturbances and those responsible for their care. Sleep fragmentation and inadequate sleep quality can potentially worsen behavioral and cognitive difficulties, hence diminishing overall quality of life [9]. Respiratory disruption, including severe apneas and hypoventilation, has been linked to inferior long-term results in persons diagnosed with epileptic encephalopathies. The occurrence of hypoxia, characterized by reduced levels of oxygen, as a consequence of apneas, and hypercapnia, characterized by excessive CO_2_ levels, due to hypoventilation, has the potential to cause long-term brain damage, hence exacerbating impairments in cognitive and motor abilities [10]. Moreover, the heightened susceptibility to SUDEP among individuals in this demographic emphasizes the importance of addressing respiratory concerns as an integral component of comprehensive care [11]. In summary, it can be concluded that respiratory dysfunction is a prominent clinical characteristic in persons diagnosed with epileptic encephalopathies. The significance of acknowledging and resolving the issues associated with managing complicated illnesses is underscored by the diverse array of respiratory abnormalities identified, the reciprocal association with seizures, and the considerable impact on both quality of life and prognosis.

Mechanisms underlying respiratory dysfunction

The occurrence of respiratory failure in epileptic encephalopathies is a multidimensional phenomenon characterized by intricate underlying mechanisms. Comprehending these pathways is essential for formulating precise therapies and enhancing the comprehensive care and management of patients impacted by these illnesses. This section examines the neurological, cerebral, autonomic, and molecular mechanisms that contribute to respiratory problems observed in individuals with epileptic encephalopathies.

Neurobiological Mechanisms 

The genesis of seizures and respiratory failure in epileptic encephalopathies are closely linked to abnormalities in neurotransmitters, specifically gamma-aminobutyric acid (GABA) and glutamate. GABA serves as the primary inhibitory neurotransmitter within the brain, playing a critical role in maintaining a delicate equilibrium between excitatory and inhibitory impulses. Mutations in genes related to GABA receptors and transporters in epileptic encephalopathies might disturb the equilibrium, leading to heightened neuronal excitability [12]. As a result, this increased excitability can affect the respiratory centers in the brainstem, resulting in abnormal breathing patterns, apneas, and hypoventilation [11]. One illustrative instance involves the identification of mutations in the *GABRG2* gene, which encodes a subunit of the GABA-A receptor, as a contributing factor in the development of Dravet syndrome [13]. These genetic alterations can potentially hinder GABA's inhibitory function, increasing neuronal excitability. This heightened excitability may have implications for regulating respiration in the brainstem. Glutamate serves as the principal excitatory neurotransmitter within the central nervous system. Dysregulation of glutamatergic signaling has been implicated in the pathogenesis of epileptic encephalopathies and respiratory problems. In cases such as LGS, evidence suggests that mutations in genes responsible for generating ion channels and receptors involved in transmitting glutamatergic neurotransmitters may amplify excitatory signaling. The presence of these mutations has the potential to induce seizures and afterward affect the respiratory centers of the brainstem [14-16]. Intermittent respiratory abnormalities may be noted in these patients due to high stimulation.

Central Respiratory Centers

The central respiratory centers, situated in the brainstem, play a crucial role in generating and regulating rhythmic patterns of respiration. Disruptions within these central areas can potentially induce abnormal breathing patterns, apneas, and hypoventilation in persons diagnosed with epileptic encephalopathies. The medullary respiratory centers, namely, the ventral respiratory group (VRG) and the dorsal respiratory group (DRG) regulate the pattern and magnitude of respiration. Irregular breathing patterns can occur as a result of disruptions in these centers. Seizures have the potential to directly impact these specific centers, resulting in breathing disruptions both during and following the occurrence of seizures [13]. The VRG regulates both the expiratory and inspiratory phases of respiration. Modifications in this specific area might lead to inefficient or superficial respiratory patterns, which can be detected as erratic breathing in persons with epileptic encephalopathies [14]. The respiratory centers in the pons, specifically the pneumatic and apneustic centers, play a crucial role in regulating the shift from inspiration to expiration. Any perturbation in the precise adjustment of these centers can result in aberrant respiratory patterns. The occurrence of seizures has the potential to spread to the pontine respiratory centers, influencing their normal functioning. This phenomenon can disrupt the regular respiration pattern, hence playing a role in developing respiratory irregularities [17].

Autonomic Control

The autonomic nerve system (ANS) is essential in regulating respiratory functions, specifically concerning physiological demands and stresses. The autonomic nervous system (ANS) dysregulation can potentially worsen respiratory dysfunction in individuals with epileptic encephalopathies. The cranial nerve X, often known as the vagus nerve, is crucial in regulating several autonomic functions, such as respiration, through parasympathetic control. The presence of deviations in vagal tone can affect both the rate and pattern of respiration. Seizures and their accompanying autonomic responses can potentially disturb the regulation of the respiratory centers by the vagus nerve in persons diagnosed with epileptic encephalopathies. Transient heart rate and breathing changes might occur, especially during the ictal and postictal periods [18]. Epileptic seizures have the potential to elicit sympathetic responses, such as an elevated heart rate (tachycardia) and heightened blood pressure. These empathetic reactions have the potential to indirectly impact the respiratory centers, resulting in changes to breathing patterns. Moreover, heightened sympathetic activity during seizures may play a role in autonomic instability, which could worsen respiratory problems in persons diagnosed with epileptic encephalopathies [19].

The Molecular and Cellular Mechanisms Behind Respiratory Dysfunction

Respiratory dysfunction in epileptic encephalopathies is influenced by various variables at the molecular and cellular levels. The presence of genetic mutations in ion channels, neurotransmitter receptors, and transporters has a direct influence on neuronal excitability and neurotransmission. The occurrence of mutations in genes such as *SCN1A*, *GABRG2*, and *GRIN2A* has been associated with different forms of epileptic encephalopathies, perhaps causing disturbances in the previously mentioned neurobiological pathways [11]. Atypical levels of neural network excitability distinguish epileptic encephalopathies. These modifications can induce hypersynchronous firing and excitation propagation to brainstem respiratory areas, resulting in breathing abnormalities during seizures [20]. The function of respiratory centers can be influenced by inflammation inside the central nervous system. Respiratory control may be disrupted by inflammatory processes, which several sources, including infections, can initiate, potentially leading to breathing problems [21]. The dysregulation of multiple neurotransmitters, including but not limited to GABA and glutamate, can potentially disrupt the delicate equilibrium between excitatory and inhibitory processes in respiratory centers. The modulation of respiratory regulation may be influenced by alterations in neurotransmitters such as serotonin and acetylcholine, among others [22]. To summarize, the presence of respiratory failure in epileptic encephalopathies is a complex phenomenon that involves various aspects of neurobiology, central nervous system function, autonomic regulation, and molecular mechanisms. The irregular breathing patterns, apneas, and hypoventilation observed in affected patients can be attributed to neurotransmitter imbalances, disruptions in central respiratory centers, autonomic dysregulation, and molecular changes.

Diagnostic challenges

Diagnosing respiratory dysfunction in individuals with epileptic encephalopathies is a complex and multifaceted task, often fraught with challenges and uncertainties. This section explores the vital diagnostic challenges, including the lack of routine screening protocols, the diversity of clinical phenotypes, and the importance of comprehensive respiratory assessments, using case studies to illustrate the diagnostic dilemmas.

Lack of Routine Screening Protocols

One of the foremost challenges in diagnosing respiratory dysfunction in epileptic encephalopathies is the absence of standardized screening protocols. Unlike conditions with more established guidelines, such as asthma or sleep apnea, there is no universally accepted approach for routinely assessing respiratory function in individuals with epileptic encephalopathies. This absence of routine screening protocols can lead to delayed or missed diagnoses. Clinicians may not consider respiratory dysfunction a potential comorbidity in these patients, especially if there are no overt symptoms or the primary focus is seizure management. Consequently, individuals may experience ongoing respiratory issues without appropriate intervention.

Diverse Clinical Phenotypes

Epileptic encephalopathies encompass a spectrum of conditions, each with its unique clinical presentation. This diversity in clinical phenotypes further complicates the diagnosis of respiratory dysfunction. For example, consider the contrast between Dravet syndrome, characterized by convulsive seizures and frequent status epilepticus, and LGS, known for its diverse seizure types and cognitive impairments. The respiratory challenges in these two conditions can vary significantly. In Dravet syndrome, respiratory abnormalities, such as hypoventilation and apneas, may be more pronounced during or after convulsive seizures, making them relatively easier to detect in a clinical setting [2]. However, in LGS, the respiratory issues may be less seizure-dependent and more chronic, with continuous irregular breathing patterns and a higher risk of SUDEP [3]. As a result, clinicians must adapt their diagnostic approach to account for the specific characteristics of each epileptic encephalopathy and consider the possibility of respiratory dysfunction as part of the differential diagnosis.

Case Studies Illustrating Diagnostic Dilemmas

To illustrate the diagnostic dilemmas associated with respiratory dysfunction in epileptic encephalopathies, let's consider two hypothetical case studies:

Case study 1 - Draft syndrome: A two-year-old child presents with a history of prolonged seizures and developmental regression, consistent with Dravet syndrome. During hospitalization for status epilepticus, the child experiences postictal hypoventilation, prompting further investigation. In this case, the respiratory dysfunction is closely tied to the seizures and is more easily recognized.

Case study 2 - LGS: A seven-year-old child with LGS presents with fragmented sleep, behavioral disturbances, and cognitive decline. The child's parents report episodes of irregular breathing during sleep, but these are not consistently observed during clinical assessments. The child's respiratory dysfunction, characterized by continuous irregular breathing patterns, is less directly linked to seizures and poses a diagnostic challenge. These case studies highlight the variability in clinical presentation and the need for comprehensive evaluations encompassing seizure-related and chronic respiratory issues. Diagnostic assessments should include continuous monitoring during sleep and daytime assessments to capture the full spectrum of respiratory abnormalities.

Importance of Comprehensive Respiratory Assessments

Given the diagnostic challenges associated with respiratory dysfunction in epileptic encephalopathies, comprehensive respiratory assessments are crucial. These assessments should be tailored to the individual's clinical presentation and history, including the type and frequency of seizures, cognitive function, and sleep patterns [23]. Polysomnography (PSG) is a valuable tool for assessing sleep-related respiratory disturbances, including apneas, hypoventilation, and irregular breathing. Continuous monitoring during sleep can reveal subtle abnormalities that may not be apparent during routine clinical evaluations. Since respiratory dysfunction may not be limited to sleep, daytime monitoring is equally important. Ambulatory monitoring devices can capture respiratory parameters during waking hours, providing a more comprehensive assessment. In cases where respiratory dysfunction is closely linked to seizures, neurophysiological assessments, such as electroencephalography (EEG), can help identify seizure-related respiratory patterns and guide management strategies [24]. Given the complex interplay between epilepsy, cognition, and respiratory function, a multidisciplinary approach involving neurologists, pulmonologists, sleep specialists, and neuropsychologists is often necessary to reach a comprehensive diagnosis and develop tailored interventions. In conclusion, diagnosing respiratory dysfunction in individuals with epileptic encephalopathies is challenging due to the lack of routine screening protocols, the diversity of clinical phenotypes, and the complex interplay between seizures and respiratory issues. Comprehensive assessments, including PSG, daytime monitoring, neurophysiological evaluations, and a multidisciplinary approach, are essential for accurately diagnosing and managing respiratory dysfunction in this population.

Therapeutic approaches

The presence of respiratory impairment in persons diagnosed with epileptic encephalopathies is a notable clinical obstacle. The effective management of intricate respiratory conditions necessitates a comprehensive strategy including the administration of AEDs, utilization of various ventilatory support modalities, investigation into developing therapeutic interventions and neuromodulation approaches, as well as the indispensable collaboration of a multidisciplinary healthcare team.

The Role of AEDs in the Management of Respiratory Dysfunction

AEDs serve as the fundamental component in the management of epileptic encephalopathies. The major objective of AEDs is to regulate seizures. However, it is worth noting that their utilization can have an indirect influence on respiratory function. This is achieved by diminishing the occurrence and intensity of epileptic episodes, which are frequently associated with disruptions in breathing patterns [25]. One of the primary factors to take into account when utilizing AEDs for the management of respiratory dysfunction is the effectiveness of these medications in controlling seizures, particularly convulsive seizures. By effectively managing seizures, AEDs can play a crucial role in mitigating the immediate effects of seizures on respiratory function. Pharmaceutical agents such as valproic acid, clobazam, and stiripentol have been employed in the treatment of distinct epileptic encephalopathies, such as Dravet syndrome, to diminish the occurrence of seizures and mitigate the concomitant respiratory difficulties [2]. Certain AEDs possess potential adverse effects that may impact respiratory function. For instance, several AEDs, such as topiramate and acetazolamide, have been associated with the occurrence of metabolic acidosis. This condition has the potential to worsen respiratory depression in persons who already have tendencies toward hypoventilation [26]. The selection of an AED should be personalized, taking into consideration the particular epileptic encephalopathy, the patient's reaction, and the existence of comorbidities, such as respiratory failure. It is imperative to engage in consistent monitoring to assess both the efficacy of seizure control and the presence of any potential adverse effects.

The Various Options for Providing Ventilatory Support

In situations when there is significant impairment of respiratory function, particularly when patients are susceptible to low oxygen levels and high CO_2_ levels, a range of ventilatory support strategies may be utilized to guarantee sufficient oxygenation and ventilation. The potential alternatives can encompass noninvasive ventilation (NIV), including bilevel positive airway pressure (BiPAP) and continuous positive airway pressure (CPAP), which has been shown to have potential benefits for persons who experience hypoventilation or have components of obstructive sleep apnea (OSA). The maintenance of open airways and the support of sufficient ventilation during sleep have been shown to reduce the likelihood of respiratory disturbances [24]. In instances of pronounced respiratory dysfunction, particularly in patients with advancing encephalopathies or those susceptible to abrupt respiratory failure, the utilization of invasive mechanical ventilation via endotracheal intubation may become necessary. The utilization of this method guarantees meticulous regulation of ventilation settings and oxygenation (25). Phrenic nerve stimulation is an example of an emerging technology that presents a neuromodulation-based method for providing respiratory assistance. Using activating the phrenic nerves, which are responsible for regulating the diaphragm, this particular treatment has the potential to assist those who experience compromised diaphragmatic function in attaining enhanced ventilation [27].

Exploration of Emerging Therapies and Neuromodulation Techniques

Ongoing research is being conducted to explore innovative therapeutics and neuromodulation techniques aimed at effectively treating respiratory dysfunction in individuals with epileptic encephalopathies. Several promising ways have been identified, including The field of gene therapy has promise in its ability to target the fundamental genetic abnormalities that play a role in the development of epilepsy and respiratory failure. Preclinical investigations examining the efficacy of gene substitution or gene editing methodologies have exhibited encouraging results in animal models, hence potentially providing prospective therapeutic avenues in the future [28]. Neuromodulation techniques, such as vagus nerve stimulation (VNS) and responsive neurostimulation (RNS), are predominantly employed for seizure management. However, it is worth noting that these approaches may also yield secondary advantages in terms of respiratory function. The investigation of these techniques is focused on their potential broader impact on neural networks and autonomic regulation, which may have implications for respiratory centers [29]. Current research is dedicated to the advancement of pharmaceutical therapies that specifically address respiratory dysfunction. The objective of these interventions is to regulate neuronal pathways and neurotransmitter systems that are implicated in the control of respiration, with the potential to provide more precise and efficient therapeutic approaches [30].

Multidisciplinary Approach to Treatment

The effective management of respiratory dysfunction in persons with epileptic encephalopathies requires the implementation of a comprehensive and interdisciplinary approach. To deliver comprehensive care, a multidisciplinary team of healthcare specialists specializing in neurology, pulmonology, sleep medicine, neuropsychology, and genetics must engage in collaborative efforts. Neurologists assume a pivotal role in the diagnosis and treatment of epileptic encephalopathies, while also being responsible for the prescription of AEDs to effectively manage seizures. It is imperative to closely observe the influence of seizures on respiratory function and subsequently modify treatment measures in response. Pulmonologists play a crucial role in the evaluation and treatment of respiratory disorders. These professionals possess the knowledge and skills necessary to proficiently diagnose and treat hypoventilation, sleep-disordered breathing, and several other respiratory ailments. Additionally, they are adept at guiding the appropriate utilization of ventilatory support alternatives. Sleep experts can perform thorough evaluations of sleep, which may involve the use of polysomnography, to diagnose breathing abnormalities that are related to sleep and customize interventions to enhance both the quality of sleep and respiratory function. Neuropsychologists can assess the cognitive and behavioral dimensions of persons diagnosed with epileptic encephalopathies, thereby contributing to the understanding and management of the combined effects of epilepsy and respiratory dysfunction on general well-being and quality of life. Genetic counselors possess the ability to offer genetic testing and counseling services to facilitate therapeutic decision-making and evaluate the likelihood of epilepsy and respiratory dysfunction in relatives, considering the hereditary underpinnings of certain epileptic encephalopathies [31]. In summary, the management of respiratory dysfunction in individuals with epileptic encephalopathies necessitates a comprehensive strategy encompassing the administration of AEDs, utilization of various ventilatory support methods, investigation of emerging therapeutic approaches and neuromodulation techniques, and the indispensable collaboration of a multidisciplinary healthcare team. Individuals diagnosed with epileptic encephalopathies can have enhanced overall health and quality of life through the simultaneous management of seizures and respiratory difficulties.

Clinical outcomes and prognosis

Respiratory dysfunction in epileptic encephalopathies carries significant long-term implications, profoundly influencing patient outcomes. Understanding the factors that shape these outcomes is vital for tailoring interventions and improving the quality of life for affected individuals. This section explores the long-term implications of respiratory dysfunction and the factors that influence patient outcomes and provides case studies to illustrate the impact on prognosis.

Long-Term Implications of Respiratory Dysfunction in Epileptic Encephalopathies

Respiratory dysfunction, ranging from intermittent apneas to chronic hypoventilation, can significantly diminish the quality of life for individuals with epileptic encephalopathies. Sleep disturbances, daytime fatigue, and the need for ventilatory support devices can limit their ability to engage in typical daily activities and social interactions [32]. Chronic respiratory disturbances can exacerbate cognitive and behavioral challenges in epileptic encephalopathies. Poor sleep quality resulting from respiratory dysfunction can lead to cognitive decline and behavioral problems, compromising patient outcomes [33]. Perhaps the most concerning long-term implication is the increased risk of mortality associated with respiratory dysfunction. Individuals with epileptic encephalopathies, especially those with severe forms like LGS, have a heightened risk of SUDEP [34]. Respiratory dysfunction, often exacerbated during seizures, is pivotal in this risk.

Factors Influencing Patient Outcomes

Timely diagnosis and intervention are crucial for improving patient outcomes. Early recognition of respiratory dysfunction, followed by appropriate therapeutic measures, can mitigate its impact on quality of life and prognosis [35]. Effective seizure control through AEDs is closely linked to better respiratory outcomes. Seizures, especially convulsive seizures, can directly exacerbate respiratory dysfunction, so controlling them can minimize the immediate risk [36]. The presence of comorbidities, such as cognitive impairment or cardiac abnormalities, can complicate the management of respiratory dysfunction and influence patient outcomes. Comprehensive care that addresses these comorbidities is essential [32].

Case Studies Demonstrating the Impact on Prognosis

Case study 1 - LGS: A 10-year-old child diagnosed with LGS exhibits severe cognitive impairments, multiple seizure types, and respiratory disturbances, including intermittent hypoventilation during sleep. Despite efforts to manage seizures with AEDs, the child's respiratory function continues deteriorating. Frequent respiratory infections and hospitalizations further compromise the prognosis. In this case, respiratory dysfunction, exacerbated by seizures, contributes to a challenging clinical course. The multidisciplinary team focuses on optimizing respiratory support, including NIV during sleep, to improve the child's quality of life and mitigate the risk of SUDEP.

Case study 2 - Draft syndrome: A 5-year-old child with Dravet syndrome experiences frequent seizures and prolonged episodes of status epilepticus. During these episodes, the child's respiratory function becomes severely compromised, leading to postictal hypoventilation. Despite attempts to control seizures with multiple AEDs, the child's prognosis remains guarded due to the life-threatening nature of the seizures. This case underscores the critical relationship between seizures and respiratory dysfunction in certain epileptic encephalopathies. In such cases, achieving seizure control is paramount to improving respiratory outcomes and overall prognosis.

Future directions and research opportunities

The management of respiratory dysfunction in epileptic encephalopathies is continuously evolving, with promising avenues for targeted therapies, predictive biomarkers, advanced monitoring technologies, and collaborative research efforts on the horizon.

Potential for Targeted Therapies

Advances in gene therapy hold promise for addressing the underlying genetic mutations that contribute to epilepsy and respiratory dysfunction. Tailored gene-editing techniques may provide a path to targeted treatments [36]. Ongoing research aims to identify pharmacological agents that can modulate the neural pathways and neurotransmitter systems involved in respiratory control. These agents may offer more targeted and effective therapies [34].

Development of Predictive Biomarkers

Genetic markers associated with respiratory dysfunction susceptibility are being explored. Identifying genetic variants that increase the risk of respiratory abnormalities can guide early intervention and personalized treatment strategies [33]. Biomarkers associated with SUDEP risk, including autonomic dysfunction and respiratory control markers, are under investigation. Finding reliable biomarkers could help identify individuals at higher risk and guide preventive measures [31].

Integration of Advanced Monitoring Technologies

Advanced neuroimaging techniques, such as functional magnetic resonance imaging (fMRI) and positron emission tomography (PET), are being employed to understand the neural mechanisms underlying respiratory dysfunction better. This knowledge can inform targeted interventions [29]. Portable monitoring technologies that allow continuous tracking of respiratory parameters, including oxygen saturation and ventilatory patterns, are being developed. These tools enable real-time assessment and intervention [28].

Collaborative Efforts in Research and Innovation

Collaborative efforts among multidisciplinary researchers, clinicians, and institutions are essential for advancing our understanding of respiratory dysfunction in epileptic encephalopathies. Combining expertise from neurology, pulmonology, genetics, and other fields can accelerate progress [29]. Conducting well-designed clinical trials to evaluate novel therapeutic approaches is critical. These trials should incorporate comprehensive respiratory assessments and consider seizure control and respiratory outcomes as primary endpoints [35]. Engaging patients and their families in research efforts is invaluable. Their experiences and insights can inform the development of patient-centered interventions and research priorities [36]. In conclusion, the long-term implications of respiratory dysfunction in epileptic encephalopathies are substantial, affecting the quality of life, cognitive and behavioral outcomes, and even mortality risk. Early diagnosis, effective seizure control, and the management of comorbidities are essential factors influencing patient outcomes [37-40]. Future directions in research offer promising opportunities for targeted therapies, predictive biomarkers, advanced monitoring technologies, and collaborative efforts to enhance the management and prognosis of respiratory dysfunction in this complex population.

## Conclusions

In epileptic encephalopathies, the relationship between seizures and respiratory dysfunction has risen to the vanguard of clinical consideration. This narrative review has shed light on several crucial insights and challenges about respiratory dysfunction in this complex context. First, respiratory dysfunction in epileptic encephalopathies has far-reaching effects, affecting not only the immediate health of affected individuals but also their long-term quality of life and, in some cases, their very survival. Timely diagnosis, seizure control, and the management of comorbidities emerge as significant determinants of patient outcomes. In addition, the multidisciplinary approach, which includes neurologists, pulmonologists, sleep specialists, and genetic counselors, is essential for providing comprehensive treatment to these patients. This comprehensive approach addresses the complex relationship between epilepsy and respiratory disorders. However, obstacles remain, such as the requirement for targeted therapies, identifying predictive biomarkers, and incorporating advanced monitoring technologies. Collaboration across disciplines is essential for advancing our knowledge and improving patient care. This review concludes by emphasizing the complexity of respiratory dysfunction in epileptic encephalopathies and the critical need for continued research and innovation. By confronting these obstacles head-on, we can improve the lives of those affected by these conditions and offer hope for a brighter future.
